# Role of extracorporeal membrane oxygenation in children with sepsis: a systematic review and meta-analysis

**DOI:** 10.1186/s13054-020-03418-z

**Published:** 2020-12-07

**Authors:** Kollengode Ramanathan, Nicholas Yeo, Peta Alexander, Lakshmi Raman, Ryan Barbaro, Chuen Seng Tan, Luregn J. Schlapbach, Graeme MacLaren

**Affiliations:** 1 Cardiothoracic Intensive Care Unit, Department of Cardiothoracic Surgery, National University Hospital, Singapore, 119228 Singapore; 2 Yong Loo Lin School of Medicine, National University of Singapore, Singapore, Singapore; 3 Queen’s University Belfast School of Medicine, Belfast, UK; 4 Department of Cardiology, Boston Children’s Hospital, Boston, MA USA; 5 Department of Pediatrics, Harvard Medical School, Boston, MA USA; 6 University of Texas Southwestern Medical Center, Dallas, USA; 7 Division of Pediatric Critical Care Medicine, University of Michigan, Ann Arbor, USA; 8 Department of Intensive Care Medicine and Neonatology, and Children’s Research Center, University Children’s Hospital of Zurich, University of Zurich, Zurich, Switzerland; 9 Pediatric Critical Care Research Group, Child Health Research Centre, The University of Queensland and Queensland Children’s Hospital, Brisbane, QLD Australia

**Keywords:** Pediatric, Neonatal, Sepsis, Septic shock

## Abstract

**Background:**

The benefits of extracorporeal membrane oxygenation (ECMO) in children with sepsis remain controversial. Current guidelines on management of septic shock in children recommend consideration of ECMO as salvage therapy. We sought to review peer-reviewed publications on effectiveness of ECMO in children with sepsis.

**Methods:**

Studies reporting on mortality in children with sepsis supported with ECMO, published in PubMed, Scopus and Embase from 1972 till February 2020, were included in the review. This study was done in adherence to Preferred Reporting Items for Systematic Review and Meta-Analysis statement after registering the review protocol with PROSPERO. Study eligibility was independently assessed by two authors and disagreements resolved by a third author. Publications were reviewed for quality using Grading of Recommendations Assessment, Development, and Evaluation (GRADE) system. Random-effects meta-analyses (DerSimonian and Laird) were conducted, and 95% confidence intervals were computed using the Clopper-Pearson method. Outliers were identified by the Baujat plot and leave-one-out analysis if there was considerable heterogeneity. The primary outcome measure was survival to discharge. Secondary outcome measures included hospital length of stay, subgroup analysis of neonatal and paediatric groups, types and duration of ECMO and complications .

**Results:**

Of the 2054 articles screened, we identified 23 original articles for systematic review and meta-analysis. Cumulative estimate of survival (13 studies, 2559 patients) in the cohort was 59% (95%CI: 51–67%). Patients had a median length of hospital stay of 28.8 days, median intensive care unit stay of 13.5 days, and median ECMO duration of 129 h. Children needing venoarterial ECMO (9 studies, 208 patients) showed overall pooled survival of 65% (95%CI: 50–80%). Neonates (< 4 weeks of age) with sepsis needing ECMO (7 studies, 85 neonates) had pooled survival of 73% (95%CI: 56- 87%). Gram positive organisms were the most common pathogens (47%) in septic children supported with ECMO.

**Conclusion:**

Survival rates of children with sepsis needing ECMO was 59%. Neonates had higher survival rates (73%); gram positive organisms accounted for most common infections in children needing ECMO. Despite limitations, pooled survival data from this review indicates consideration of ECMO in refractory septic shock for all pediatric age groups.

## Background

Sepsis and septic shock remain an important cause of childhood mortality globally despite advances in early detection and appropriate management in Intensive Care Units (ICUs) [1]. The World Health Assembly, the decision-making body of the World Health Organisation, adopted a recent resolution to reduce the global burden of sepsis through early identification, prevention and management [1–5]. The percentage of all global deaths in 2017 related to sepsis was highest in neonates and children compared to the adult population [5]. A recent systematic review revealed that nearly 1 in 4 children with sepsis die. Children most at risk of death are younger patients and those with septic shock; sepsis and infections account for 6.3 deaths of 1000 live births among children younger than 5 years [6].

While current best practice includes the early delivery of sepsis treatment bundles, therapeutic options for children refractory to initial resuscitation remain very limited [7]. The development of refractory shock is associated with a steep increase in mortality in pediatric sepsis [8–11]. Previous sepsis guidelines, including the American College of Critical Care Medicine, recommended consideration for Extracorporeal Membrane Oxygenation (ECMO) for persistent shock in spite of early fluid- and inotrope-based resuscitation [12, 13]. Studies have reported an increase in use of ECMO for pediatric septic shock with improving outcomes over time [14–17]. Survival rates of septic patients supported with ECMO are above 70% for neonates [16, 18, 19] and approximately 40% for older children in both single centre and registry studies. Accordingly, the 2020 Surviving Sepsis Campaign International Guidelines for the Management of Septic Shock and Sepsis-Associated Organ Dysfunction in Children recommended venoarterial ECMO as rescue therapy in children with septic shock only if refractory to all other treatments, with a low level of evidence and a weak recommendation [20].

These recommendations remain based on few, relatively small studies. Additional challenges relate to the difficulties in assessing outcomes in heterogeneous cohorts of critically ill children and the number of factors which may contribute to outcomes, including patient selection, time to ECMO initiation, cannulation strategies, different ECMO technology, and the experience of the treating team [17, 21]. As a result, the outcome benefits of ECMO in pediatric sepsis remain controversial. We sought to systematically review the literature to examine survival rates of pediatric and neonatal patients with sepsis needing ECMO, and to describe etiologies of sepsis and the complication rates in published cohorts of neonates and children treated with ECMO for sepsis.

## Methods

This study was done in adherence to the ethical guidelines stated in the Declaration of Helsinki following the Preferred Reporting Items for Systematic Review and Meta-Analysis (PRISMA) statement. [22] The review protocol was registered with PROSPERO (Ref:CRD 42020161828).

### Study selection

A comprehensive literature search was performed using MEDLINE, Embase and Scopus databases to retrieve published data from 1972 up to 15^th^ February 2020 on the use of ECMO in neonatal and pediatric septic patients. The search phrases for the three databases included Boolean terms ‘AND’ and ‘OR’ with the following keywords in various possible combinations: “ECMO”, “extracorporeal membrane oxygenation”, “ECLS”, “Infant”, “newborn”, “neonatal”, “pediatric”, “sepsis”, and “infection” [Additional file 1: eTable 1]. In addition, a hand search of all relevant studies and their citation lists was performed to identify additional articles for inclusion. Articles were selected for systematic review if they described neonatal and/or pediatric septic patients undergoing venoarterial and venovenous ECMO and the incidence of mortality was clearly stated. No restriction was placed on study type (prospective or retrospective). For studies that included overlapping patients (period of overlap > 1 year), the largest study was included in the meta-analysis while the rest of the studies were included in the review. We excluded studies with predominantly patients above 18 years old, articles with fewer than 5 patients, articles not written in English, conference abstracts, surveys and articles without full text, review articles and case reports. Publications from the Extracorporeal Life Support Organization (ELSO) International registry were excluded since data on patients reported to the ELSO Registry are likely also reported to national databases and present in single center reviews and thus potentially duplicated. Studies based on national registers were included provided there was no overlap with single-center studies. The eligibility of studies was independently assessed by two review authors (NY and KR) and disagreements were resolved by consensus or appeal to a third author (GM). Publications were reviewed for quality using the Joanna Briggs Institute (JBI) checklist for prevalence studies [23] as well as the Grading of Recommendations Assessment, Development, and Evaluation (GRADE) system to the determine the overall rating confidence in the body of evidence [24]. Case reports and case series (< 5 patients) were excluded due to likelihood of positive outcome bias.

### Study analysis

Data including study design, outcomes, patient characteristics, and interventions were extracted independently. Neonates were defined as children 28 days or less of age. Descriptive statistics, such as the medians and 25th to 75th percentiles were reported for continuous variables, and the counts and percentages were reported for categorical variables. Survival to discharge was the primary measure of outcome in our meta-analysis. Secondary outcome measures included hospital length of stay, types and duration of ECMO and complications. Subgroup analysis were also performed for the neonatal and pediatric age group.

We anticipated heterogeneity between studies. To account for the variability between studies in the meta-analysis, we used the random-effects model that was based on the inverse variance method where the DerSimonian and Laird method [25] was used to estimate the between-study variance. Briefly for the meta-analysis of proportions, exact confidence interval (CI) for each proportion was computed using the Clopper-Pearson method [26]. A variance-stabilizing transformation, called the Freeman-Tukey double arcsine transformation [27], was applied to all proportions and the meta-analysis was performed on the transformed proportions. The pooled transformed proportion and its 95%CI were back transformed to obtain pooled estimates on the original proportion scale. To obtain a pooled mean age of study participants across studies, studies that did not report the mean and/or its corresponding standard deviation (SD) had these quantities estimated from the reported medians, range, and IQR using the methods proposed by Wan et al. [28].

Statistical heterogeneity between studies was identified using I^2^ statistics, where I^2^ ≤ 40%, between 30–60%, between 50–75% and ≥ 75% indicated low, moderate, substantial, and considerable heterogeneity, respectively. P values for I^2^ statistic were derived from the chi-square distribution of Cochran’s Q test. Together with the visual inspection of the forest plot, additional diagnostics were carried out to identify outlying or/and influential studies. Firstly, studies with studentized deleted residuals greater than 1.96 or less than -1.96 were identified as potential outliers [29]. Next, a set of leave-one-out analysis was performed to identify potential influential studies that resulted in a large change in the pooled estimates after they were left out one at a time from all studies. Lastly, a Baujat plot was used to identify studies that had high contributions to the heterogeneity in the meta-analytic data [30]. With these diagnostics, an agreement was made between two investigators to identify and remove outlying/influential studies. A funnel plot and Egger’s tests was conducted to assess for reporting publication bias. Univariate meta regression analysis was also conducted on pre-ECMO characteristics where at least 4 data points were available. The analyses were conducted using the R Studio (Version 3.6.1, R Studio, Inc. Boston) using the ‘metafor’ and ‘meta’ packages.

### Results

Of the 2054 articles screened, we identified 23 observational studies for systematic review and meta-analysis [3, 14, 15, 17, 31–48] (Table 1 and Additional file 1: Fig. 1); fifteen articles had overlapping information. Ten articles (6 from Australia, 1 from UK, 1 from Taiwan, 1 from USA, and 1 Multi regional) were excluded from the meta-analysis to best represent the population in terms of the period of study and setting, with minimal data loss and overlap. The definition of sepsis and septic shock was variable; most centers followed institutional or international guidelines to define criteria for ECMO initiation. Characteristics of the 13 retrospective studies included in the meta-analysis are summarized in Additional file 1: eTables 2, 3, and 4. The number of patients ranged from 7 to 1358 per study.

**Table 1:** List of 23 articles included in systemic review and meta-analysis that reported on children with sepsis needing ECMO

S2tudy author	Year	Sample Size	Age (mean/median), (IQR, SD)	% male	Type of ECMO	Country/location
C. W. Lillehei et al	1989	8	Not Stated	Not Stated	Not Specified	USA (Children’s Hospital Boston)
S. McCune et al	1990	10	80hrs (37)	Not Stated	10 VA	USA (Children’s National Medical Center)
J. R. Hocker et al	1992	15	Not Stated	Not Stated	Not Specified	USA (Kosair Children’s Hospital)
D. Cochrane et al	1992	5	Not Stated	Not stated	5 VA	Australia (The Royal Children’s Hospital)
M. Nagaya et al	1993	7	0.74y (0.7–0.8)	Not Stated	7 VA	Japan (Central Hospital, Aichi)
J. Beca and W. Butt	1994	9	12y (0.2–15)	56	9 VA	Australia (The Royal Children’s Hospital)
A.P. Goldman et al	1997	12	Not Stated	Not Stated	10 VA, 2VV	Multi-center (Australia and UK)
D. K. Luyt et al	2004	11	1y (0.8–1.3)	Not Stated	7 VA, 4VV	UK (Glenfield Hospital, Leicester)
G. MacLaren et al	2007	45	2.5y (0.4–9)	62	45 VA	Australia (The Royal Children’s Hospital)
R. Tiruvoipati et al	2007	6	4y (0.17–14)	Not stated	2 VA, 4 VV	UK (Glenfield Hospital, Leicester)
S. J. Wu et al	2007	8	4.3y (2.9–63)	50	5 VA, 3 VV	Taiwan (Mackay Memorial Hospital)
S. Horton et al	2010	47	1.4y (0.1–7.5) 3.9y (0.5- 12.8)	63 67	35 Peripheral VA 12 Central VA	Australia (The Royal Children’s Hospital)
G. MacLaren et al	2011	23	6y (2.8–12.3)	57	23 Central VA	Australia (The Royal Children’s Hospital)
C. C. Peng et al	2012	12	4.7y (3.1)	58	8 VA, 4VV	Taiwan (Mackay Memorial Hospital)
Y. Kawai et al	2015	14	12y (4.2–15)	64	13 VA, 1 VV	USA (University of Michigan)
J. Rambaud et al	2015	22	2.5y (3.1)	64	22 VA	France (Armand-Trousseau Hospital)
A. Ruth et al	2015	1,858	1.1y (0.2–6.7)^1^	53	Not Specified	USA (Pediatric healthcare information system)
K. Y. Chen et al	2016	7	3.9y (0.4–12.8)^2^ Not Stated	Not Stated	Not Stated	Australia (Royal Children’s Hospital and Monash Medical Center)
A. Sole et al	2018	21	3.3y (0.7–4.7) P 1 day (1–5) N	62	21 VA	Spain (Hospital Sant Joan de Deu)
F. Oberender et al	2018	44	3.6y (0.5–8.0)	52	44 VA	Australia, New Zealand, Netherlands, UK, USA
T. H. Chang et al	2018	55	7.2y (6.2)	53	Not Specified	Taiwan (National Taiwan University Children’s Hospital)
K. Robb et al	2019	415	Not Stated	Not stated	Not specified	USA (National Inpatient Sample)
L.J. Schlapbach et al	2019	80	1.3y (0.1–7.0)	61	23 Peripheral VA	Australia & New Zealand (ANZPIC Registry)
					57 Central VA	

Abbreviations: VA: Venoarterial, VV: Venovenous, Peripheral and Central refers to the type of cannulation for ECMO. P: pediatric Patients, N: Neonates, Data presented as mean ± SD, median (25th to 75th percentile), or median [range]. 1: Patients with ECMO-Only. 2 Patients with ECMO + RRT

**Fig. 1:**
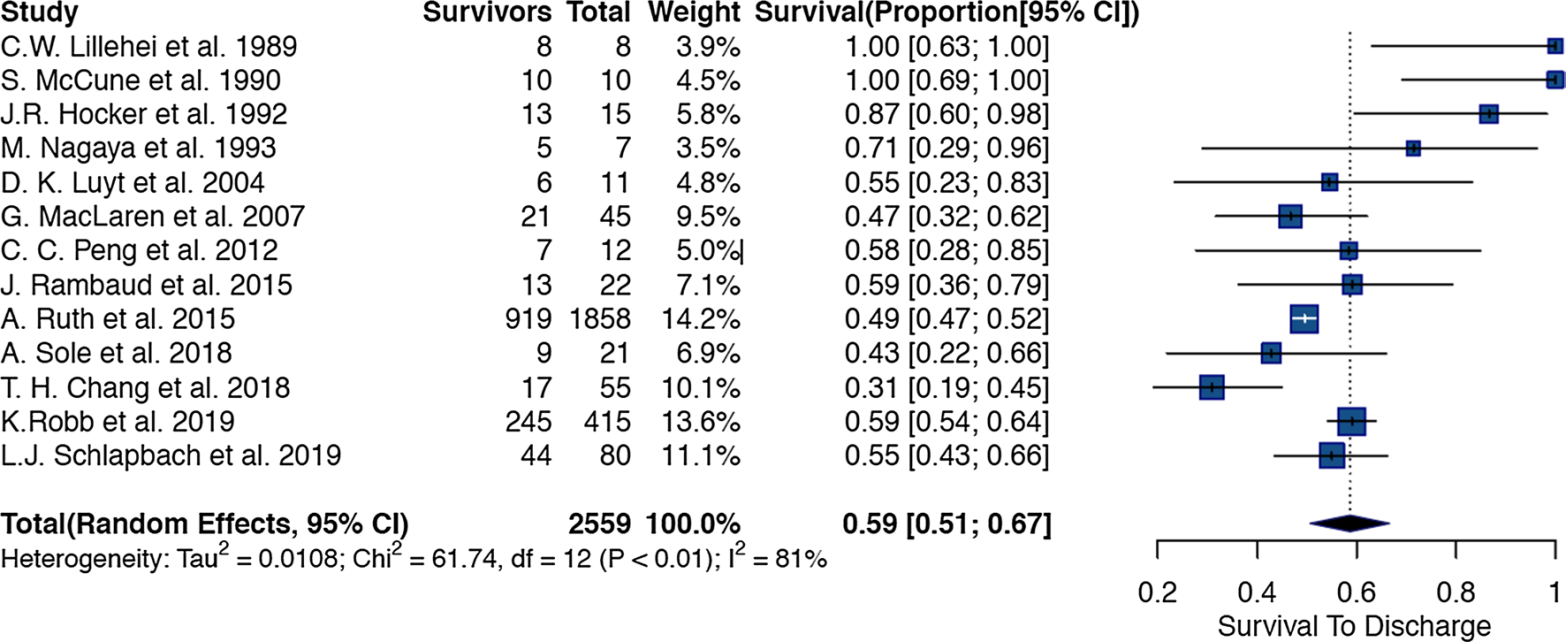
Forest plot of studies reporting on use of ECMO in children with sepsis

#### Primary outcome

Thirteen studies (Additional file 1: eTable 2) reported 2559 paediatric and neonatal patients with sepsis who needed ECMO support. The overall pooled estimate of survival in the cohort was 59% (95%CI: 51% to 67%, p < 0.01) (Fig. 1). There was considerable heterogeneity amongst the studies; we identified three studies [31, 32, 46] that were outliers or influential with the leave one out analysis and the Baujat plot. After excluding the three studies, the remaining 10 studies had 2486 patients with a cumulative pooled estimate survival of 55% (95% CI 49% to 62%, p < 0.01) (Additional file 1: Fig. 2).

**Fig. 2:**
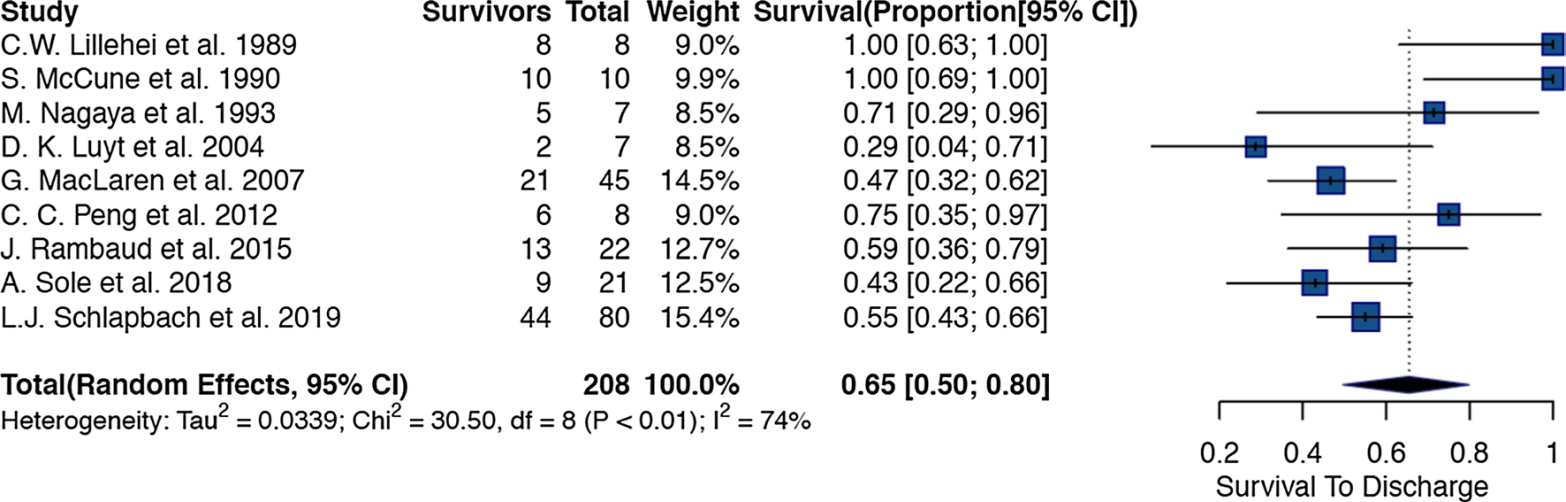
Forest plot of studies reporting on use of Venoarterial ECMO in children with sepsis

#### Secondary outcomes

Children (0–18 years) with sepsis needing ECMO support had a median length of hospital stay of 28.8 days (IQR: 13–35.5 days), a median ICU stay of 13.5 days (IQR: 9.75–26.3 days), and a median ECMO duration of 129 h (IQR: 86.3–203.8 h). Three studies reported on the duration of ICU care prior to ECMO initiation which was 34 h (IQR: 8–96), 12 h (SD 13.4) and 7 h, respectively. Two studies [15, 44] reported on duration of septic shock before ECMO which was 29.5 h (IQR: 20–46) and 22 h (IQR: 6.5–38) respectively.

#### Subgroup analysis

The pooled survival in each region was 73% (95% CI: 60% to 85%, p < 0.01) (n = 2306) in North America, 48% (95% CI: 36% to 61%, p = 0.04) (n = 199) in Australasia and 52% (95% CI: 38% to 66%, p = 0.58) (n = 54) in Europe. (Additional file 1: Figs. 3,4,5).

**Fig. 3:**
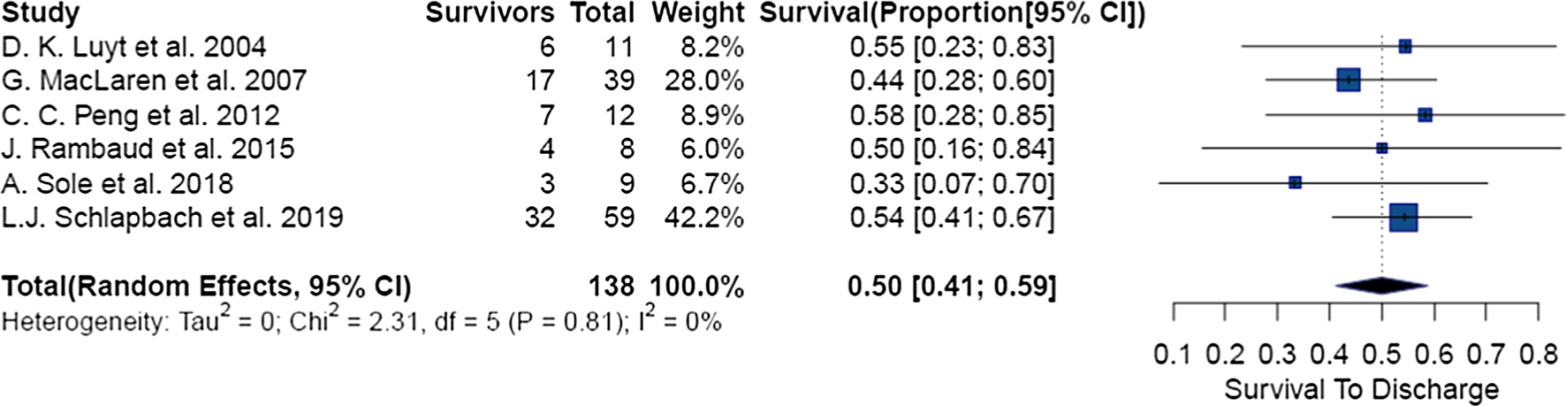
Forest plot of Pediatric group of patients needing ECMO in sepsis

Use of venoarterial ECMO was identified in 9 studies with 208 patients (95% CI: 50% to 80%, p < 0.01) and a cumulative survival of 65%. (Fig. 2) 9 of these patients were initially started with venoarterial ECMO and had been switched to venovenous ECMO during the ECMO run. After removal of 2 potential outliers [31, 32], analysis of the remaining 7 studies yielded a pooled survival rate of 53% (95%CI: 45% to 60%, p = 0.46). (Additional file 1: Fig. 6).

6 studies presented data on 138 pediatric patients aged between 28 days and 18 years with an overall pooled survival of 50% (95% CI: 41% to 59%, p = 0.81) (Fig. 3). 7 studies reported on 85 neonates with septic shock needing ECMO; overall pooled survival rate was 73% (95% CI: 56% to 87%, p = 0.03). After removal of 1 outlier study [32], 6 studies reported on 75 neonates with an overall pooled survival of 66% (95% CI: 54% to 77%, p = 0.41) (Additional file 1: Fig. 7a and b).

Univariate meta regression analysis was also conducted on pre-ECMO characteristics where at least 4 data points were available: there was no significant association found between survival and pre-ECMO lactate levels, Cardiac Arrest (%), need for renal replacement therapy (%), ECMO duration, overall length of stay in hospital and overall length of stay in ICU. (Table 2) (Additional file 1: Figs. 8–13).

**Table 2:** Meta-regression table of Potential Modifiers

Risk factors studied	No. of studies	P-value	regression coefficient [β]
Hospital Length of Stay	5	0.87	− 0.0009
ICU Length of Stay	6	0.73	− 0.0009
*Pre-ECMO Factors*			
CPR	5	0.51	− 0.0025
% Renal Replacement Therapy	7	0.77	0.0003
Lactate	4	0.89	− 0.0019
*ECMO Factors*			
ECMO duration	8	0.96	0.022

Data on use of central ECMO and survival to discharge was reported in 3 studies from Australia/New Zealand. Horton et al. (n = 12), MacLaren et al. (n = 23) and Schlapbach et al. (n = 57) reported a survival rate of 75%, 73% and 61% respectively for pediatric patients with septic shock who had atrio-aortic cannulation. The papers published by Horton et al. and MacLaren et al. were from the same institution with slightly overlapping time periods.

#### Complications

A total of 23 studies (Additional file 1: Table 2) which included overlapping studies and studies included in the meta-analysis were used in descriptive analysis of other outcomes. 14 studies presented data on complications from a total of 246 patients. The pooled rate of complications was 31% (95% CI: 19% to 44%); out of 67 reported complications, 40 (60%) were hemorrhagic, 14 (21%) were neurologic, 8 (12%) were vascular, 3 (5%) were renal, 1 (1%) was cardiovascular and 1 (1%) was infectious. 6 studies reported data on mechanical and circuit complications (e.g. circuit clotting, oxygenation failures, pump and heat exchanger malfunction and air in circuits) with a pooled complication rate of 46% (95% CI: 31%–62%).

#### Microbiological etiology

13 studies (n = 316) reported on the microbiological etiology of sepsis. Out of the 258 organisms cultured, gram positive organisms accounted for 47% (n = 121), followed by gram negative organisms at 38% (n = 99), viral infections at 14% (n = 35) and fungal infections 1% (n = 3). *Neisseria meningitidis*, *Staphylococcus aureus*, and *Streptococcus pneumoniae* were the most common organisms isolated.

#### Risk of bias

All of the 13 studies that were assessed through the JBI checklist were good quality with minimum high score of 8/9 with no concerns on risk of bias. Egger’s test and funnel plots showed some evidence of publication bias in the total ECMO group which was resolved after removal of influential studies (Additional file 1: Fig. 14). There was no evidence of publication bias in the VA-ECMO, pediatric ECMO and neonatal ECMO groups: Additional file 1: Figs. 15, 16, 17 respectively) (Additional file 1: eTable 5). The GRADE system showed high level of certainty in the use of ECMO in pediatric and neonatal and venoarterial ECMO groups and a moderate level of certainty in the overall use of ECMO in children with sepsis (Table 3).

**Table 3:** Qualitative assessment of results of metanalysis for use of ECMO in children with sepsis (GRADE analysis)

№ of studies	Certainty assessment						Effect			Certainty	Importance
	Study design	Risk of bias	Inconsistency	Indirectness	Imprecision	Other considerations	№ of events	№ of individuals	Rate (95% CI)		
*Overall survival during ECMO in children with sepsis*											
13	Observational studies	Not serious	Serious ^a^	Not serious	Not serious	None	1317	2559	0.59 (0.51 to 0.67)	⊕⊕⊕О MODERATE	CRITICAL
*VA-ECMO survival in children with sepsis*											
9	Observational studies	Not serious	Not serious	Not serious	Not serious	None	118	208	0.65 (0.50 to 0.80)	⊕⊕⊕⊕ HIGH	CRITICAL
*ECMO survival in pediatric group with sepsis*											
6	Observational studies	Not serious	Not serious	Not serious	Not serious	None	69	138	0.50 (0.41 to 0.59)	⊕⊕⊕⊕ HIGH	CRITICAL
*ECMO survival in Neonatal sepsis*											
7	Observational studies	Not serious	Not serious	Not serious	Not serious	None	59	85	0.73 (0.56 to 0.87)	⊕⊕⊕⊕ HIGH	CRITICAL

^a^There was considerable amount of heterogenity of I^2^ = 62% even after statistical removal of potential outliers that could have contributed to heterogenity, likely explained by the differences in age groups( neonate Vs pediatric), sample size and variability in JBI scores

## Discussion

The use of ECMO in pediatric sepsis has increased over the past decade [49] with variable survival rates [49–51]. Our meta-analysis of observational studies showed that overall survival rates of children with sepsis treated with ECMO were 59%. Neonates had higher survival rates (73%). Gram positive organisms were the most common type of pathogen.

The hemodynamic manifestations of septic shock can vary between different age groups. While early-onset sepsis may present predominantly as right heart dysfunction from persistent pulmonary hypertension of the newborn (PPHN), older infants and young children often manifest severe left ventricular dysfunction. Older children and adults more commonly present with distributive shock [15]. The role of ECMO in the management of refractory septic shock in neonates and children stems by virtue of its ability to provide perfusion of oxygenated blood to tissues and maintain gas exchange even in refractory cardiopulmonary failure. However, the specific threshold as to when shock, or multi-organ dysfunction should be considered “refractory”, remains poorly defined. Some data indicate that venoarterial ECMO is associated with better survival than conventional therapy in children with septic shock if high ECMO flows (> 150 mL/kg/min) can be provided [20, 45]. While data on ECMO flows were not available in all the studies included in this meta-analysis, our analysis of neonatal and pediatric septic patients who needed venoarterial (VA) ECMO showed an overall pooled survival of 63%; subsequent removal of outlier studies yielded an overall pooled survival of 53%. Children who underwent central ECMO cannulation have been shown to have survival rates up to 73% in single centre studies [17]. In comparison, the ELSO registry reported survival rates for pediatric patients with sepsis supported on ECMO of 52% [49].

The American College of Critical Care Medicine guidelines for hemodynamic support of pediatric and neonatal septic shock recommends ECMO as the therapy of choice for newborn patients with refractory PPHN and sepsis with Level 1 C evidence; however the guidelines highlight an expected survival with ECMO for septic shock being no greater than 50% in children, with Level 2 C evidence for consideration of ECMO in this group [18]. The recently published Surviving Sepsis Campaign International Guidelines in children suggested venovenous ECMO for children with sepsis induced respiratory distress syndrome and refractory hypoxia as well as venoarterial ECMO as a rescue therapy in children with septic shock refractory to all other treatments. The recommendations for both above were weak based on very low quality of evidence [20]. Our meta-analysis supplement the above recommendations and showed survival rates above 50% in septic children and more than 70% in neonatal sepsis with moderate to high level of certainty in the level of evidence.

There are several limitations to the study. This review is based on non-randomized or non-propensity-matched studies, and the studies selected for analysis showed moderate heterogeneity. The challenges of interpreting the heterogeneity using I2 values has been highlighted when studies with large sample sizes result in very narrow confidence intervals. It has also been established that I2 for the pooled estimate can be high even in the presence of modest inconsistency and can be misleading [52]. Inter-center practice variability, patient selection, sample sizes and reporting patterns likely add to the heterogeneity in our cohort. The results obtained should be interpreted in light of this heterogeneity; even if the heterogeneity was accounted for via use of the random-effects model [53–55]. The diagnostic criteria of refractory septic shock varied between studies, only a handful of studies reported the use of the 2005 pediatric Sepsis Consensus Conference Criteria [56] while some reported using the International Classification of Diseases (ICD-9) to define septic shock and most of the papers diagnosed septic shock based on their institutional guidelines. An international multicentre study based on a Delphi process followed by data-driven derivation and validation defined refractory septic shock in children as the combination of severe cardiovascular dysfunction (measured by poor cardiac function and/or preceding cardiac arrest), high lactate, and high vasopressor requirements (measured by vasopressor-inotrope score) [11]. Unfortunately, the data provided in the studies included in this metanalysis would not allow to verify if the proposed refractory septic shock criteria were met. The timing of ECMO initiation in children with sepsis could not be assessed with certainty from the literature, although one recent multicentre cohort study concluded that ECMO was only of benefit if the predicted risk of death was > 50% (48). The studies included in our analysis spanned from 1992 to 2020, where sepsis diagnosis, sepsis management, ECMO technology and ECMO practice have undergone substantial changes. Also, meta-regression analyses are inherently constrained by a lack of power, resulting in an increased risk of Type 2 errors suggesting that lack of significant association does not imply that a correlation does not exist [57]. However, this remains the largest available cohort of patients which has been analysed so far; there was no evidence of publication bias and the methodological quality of all the studies included in our analysis scored high (> 8/9), using the JBI critical appraisal tool (Additional file 1: Table 6). The GRADE analysis demonstrated moderate to high certainty in the evidence presented in this paper.

## Conclusion

The use of ECMO to resuscitate and support children with sepsis and refractory shock is feasible and is associated with survival rates as high as 59%. Neonates had higher survival rates and central cannulation may be associated with better outcomes in children needing ECMO with sepsis. Despite the limitations of studies included in this metanalysis, the aggregate data indicate that survival for patients treated with septic shock overall justifies recommendation to consider ECMO in refractory septic shock for all pediatric age groups. Future studies should provide more evidence to inform on timing and thresholds for ECMO initiation, including dynamic assessment of response to therapy/deterioration.

## Supplementary information

**Additional file 1**. Supplementary file containing additional figures (Supplementary figures 1-17) andtables( Supplementary tables 1-7)

## Data Availability

Available.
