# Supplementary material for: Role of extracorporeal membrane oxygenation in children with sepsis: a systematic review and meta-analysis
Source: Crit Care. 2020 Dec 7;24:684. doi: 10.1186/s13054-020-03418-z (PMC7720382; doi:10.1186/s13054-020-03418-z)
Supplement: Supplementary file 1 — Additional file 1. Supplementary file containing additional figures (Supplementary figures 1-17) andtables( Supplementary tables 1-7) [file 13054_2020_3418_MOESM1_ESM.docx]

| Database | Search Phrase/Instructions |
| --- | --- |
| **PubMed** | ("Extracorporeal Membrane Oxygenation"[Mesh] OR ECMO[Title/Abstract] OR "Extracorporeal Membrane Oxygenation"[Title/Abstract] OR extracorporeal life support[Title/Abstract] OR extracorporeal membrane oxygenator support[Title/Abstract]) AND (Infant*[Title/Abstract] OR newborn*[Title/Abstract] OR child*[Title/Abstract] OR neonat*[Title/Abstract] OR pediatric*[Title/Abstract]) AND ("Sepsis"[Mesh] OR Sepsis[Title/Abstract] OR Infection[Title/Abstract]) |
| **Embase** | 1. exp extracorporeal oxygenation/ 2. ECMO or Extracorporeal Membrane Oxygenation or extracorporeal life support or extracorporeal membrane oxygenator support).ti. or (ECMO or Extracorporeal Membrane Oxygenation or extracorporeal life support or extracorporeal membrane oxygenator support).ab. 3. exp sepsis/s 4. (sepsis or infection).ti. or (sepsis or infection).ab. 5. (Infant* or newborn* or child* or neonat* or pediatric*).ti. or (Infant* or newborn* or child* or neonat* or pediatric*).ab.   Combine (1 OR 2) AND (3 OR 4) AND (4) |
| **Scopus** | TITLE-ABS-KEY ( ( ecmo  OR  "Extracorporeal Membrane Oxygenation"  OR  "Extracorporeal life support"  OR  "Extracorporeal membrane oxygenator support" )  AND  ( sepsis  OR  infection )  AND  ( infant*  OR  newborn*  OR  child*  OR  pediatric* ) ) |

**Supplementary Table 1**: Literature search phrases used for database extraction from Pub Med , Embase and Scopus.

| Study author | Year | Duration of Study | Age (Mean/Median), (IQR, SD) | % male | Sample Size/Type of Ecmo | Country/Location |
| --- | --- | --- | --- | --- | --- | --- |
| C. W. Lillehei et al. | 1989 | 1984 – 1988 | Not Stated | Not Stated | 8 - NS | USA (Children’s Hospital Boston) |
| S. McCune et al. | 1990 | 1984 - 1986 | 80hrs (37) | Not Stated | 10 VA | USA (Children’s National Medical Center) |
| J. R. Hocker et al. | 1992 | 1982 – 1989 | Not Stated | Not Stated | 15 - NS | USA (Kosair Children’s Hospital) |
| M. Nagaya et al. | 1993 | 1986 – 1992 | 0.74y (0.7 – 0.8) | Not Stated | 7 VA | Japan (Central Hospital, Aichi) |
| D. K. Luyt et al. | 2004 | <2004 | 0.83y (0.5 – 1.3) | Not Stated | 7 VA, 4VV | UK (Glenfield Hospital, Leicester) |
| G. Maclaren et al. | 2007 | 1988 - 2007 | 2.5y (0.4 - 9) | 62 | 45 VA | Australia (The Royal Children’s Hospital) |
| C. C. Peng et al. | 2012 | 2001 – 2009 | 4.7y (3.1) | 58 | 8VA, 4VV | Taiwan (Mackay Memorial Hospital) |
| J. Rambaud et al. | 2015 | 2004 – 2013 | 2.5y (3.1) | 64 | 22 VA | France (Armand-Trousseau Hospital) |
| A. Ruth et al. | 2015 | 2004 - 2012 | 1.1y (0.2-6.7)^1^ | 53 | 1,858 - NS | USA (Pediatric healthcare information system) |
|  |  |  | 3.9 (0.4–12.8)^2^ |  |  |  |
| A. Sole et al. | 2018 | 2001 - 2017 | 3.3y (0.7 – 4.7) **P**  1day (1-5) **N** | 62 | 21 VA | Spain (Hospital Sant Joan de Deu) |
| T. H. Chang et al. | 2018 | 2008 – 2015 | 7.2y (6.2) | 53 | 55 - NS | Taiwan (National Taiwan University Children’s |
|  |  |  |  |  |  | Hospital) |
| K. Robb et al. | 2019 | 2012 – 2014 | Not Stated | Not stated | 415 -NS | USA (National Inpatient Sample) |
| L.J. Schlapbach et al. | 2019 | 2002 - 2016 | 1.3y (0.1 – 7.0) | 61 | 23 P-VA | Australia & New Zealand (ANZPIC Registry) |
|  |  |  |  |  | 57 C-VA |  |

Supplementary Table 2: Baseline Demographics as reported in 13 Studies Included in Meta-analysis

Abbreviations: VA: Venoarterial, VV: Venovenous, P and C : Refers to Peripheral and Central cannulation for ECMO. P: Pediatric Patients, N: Neonates. ,. NS: Not Specified Data presented as mean ± SD, median (25^th^ to 75^th^ percentile), or median [range]. 1: Patients with ECMO-Only,2 Patients with ECMO+RRT

Supplementary Table 3: Patient Outcomes as reported in 13 Studies Included in Meta-analysis

| Study author | Year | Sample Size | Survival (%) | ECMO Duration (Hours) | Length of stay in hospital (days) | Length of stay in ICU (Days) | Duration with septic shock before ECMO (Hours) | Time to ECMO on admission to ICU/Duration of ICU care to ECMO (Hours) |
| --- | --- | --- | --- | --- | --- | --- | --- | --- |
| C. W. Lillehei et al. | 1989 | 8 | 8 (100) | Not Stated | Not Stated | Not Stated | Not Stated | Not Stated |
| S. McCune et al. | 1990 | 10 | 10 (100) | 167 (60) | Not Stated | Not Stated | Not Stated | Not Stated |
| J. R. Hocker et al. | 1992 | 15 | 13 (86) | Not Stated | Not Stated | Not Stated | Not Stated | Not Stated |
| M. Nagaya et al. | 1993 | 7 | 5 (71) | 93 (35 – 186) | Not Stated | Not Stated | Not Stated | Not Stated |
| D. K. Luyt et al. | 2004 | 11 | 6 (55) | 98 (74 – 165) | Not Stated | Not Stated | Not Stated | 34 (8 – 96 |
| G. Maclaren et al. | 2007 | 45 | 21 (47) | 84 (32 – 135) | 16 (3.6 - 36) | 9.0 (3.6 – 14) | 22 (6.5 – 38) | Not Stated |
| C. C. Peng et al. | 2012 | 12 | 7 (58) | 241 (24 – 689) | Not Stated | 30.2 (21.7) | Not Stated | Not Stated |
| J. Rambaud et al. | 2015 | 22 | 13 (59) | 178 (24 – 408) N  142 (72 – 240) P | Not Stated | 17.0 (4 – 40) | Not Stated | 12 (13.4) |
| A. Ruth et al. | 2015 | 1,858 | 919 (49) | Not Stated | 35 (16–67) | 25 (12–48) | Not Stated | Not Stated |
| A. Sole et al. | 2018 | 21 | 9 (43) | 84 | 10 (2.3 – 39.5) | 10.0 (2.3 – 19) | 29.5 (20 – 46) | Not Stated |
| T. H. Chang et al. | 2018 | 55 | 17 (31) | 216 | 36 (0 – 162) | Not Stated | Not Stated | Not Stated |
| K. Robb et al. | 2019 | 415 | 245 (59) | Not Stated | 28.8 (2.9) | Not Stated | Not Stated | Not Stated |
| L.J. Schlapbach et al. | 2019 | 80 | 44 (55) | Not Stated | Not Stated | 10.0 (2.5 – 18.9) | Not Stated | 7 |

Abbreviations: P: Peripheral Cannulation. C: Central Cannulation. N: Neonates. P: Pediatric Patients

Supplementary Table 4: Pre-ECMO patient characteristics in 13 Studies Included in Meta-analysis

| Study author | Year | Sample Size | | pH | PaO2 (mmHg) | PaCO2  (mmHg) | Lactate (mmol/L) | Cardiac Arrests (%) | Need for Hemofiltration/Renal Replacement Therapy  (%) |
| --- | --- | --- | --- | --- | --- | --- | --- | --- | --- |
| C. W. Lillehei et al. | 1989 | | 8 | Not Stated | Not Stated | Not Stated | Not Stated | Not Stated | Not Stated |
| S. McCune et al. | 1990 | | 10 | Not Stated | Not Stated | Not Stated | Not Stated | Not Stated | Not Stated |
| J. R. Hocker et al. | 1992 | | 15 | Not Stated | Not Stated | Not Stated | Not Stated | Not Stated | Not Stated |
| M. Nagaya et al. | 1993 | | 7 | Not Stated | Not Stated | Not Stated | Not Stated | Not Stated | 5 (71) |
| D. K. Luyt et al. | 2004 | | 11 | Not Stated | 52 (38 – 71) | Not Stated | Not Stated | Not Stated | 8 (72) |
| G. Maclaren et al. | 2007 | | 45 | 7.25 (7.14 – 7.32) | 64 (45 – 110) | 43 (32 – 51) | 8.1 (5.1 to 12.3) | 18 (40) | 28 (62) |
| C. C. Peng et al. | 2012 | | 12 | 7.30 (0.137) | 50 (12.80) | 38 (11.18) | Not Stated | Not Stated | Not Stated |
| J. Rambaud et al. | 2015 | | 22 | 7.13 (0.12) – N  6.97 (0.14) – P | Not Stated | 54 (13.55) – N  73 (41.6) - P | 7.94 (4.92) – N  5.20 (3.50) - P | 2 (9) | 4 (18) |
| A. Ruth et al. | 2015 | | 1,858 | Not Stated | Not Stated | Not Stated | Not Stated | Not Stated | 500 (27) |
| A. Sole et al. | 2018 | | 21 | 7.11 (7.00 -7.23) | 49 (31.1 – 75) | 59 (40.9 – 70) | 13.3 (5.6 – 17.8) | 11 (52) | 15 (71) |
| T. H. Chang et al. | 2018 | | 55 | 7.24 (0.19) | Not Stated | 52.2 (31.77) | Not Stated | 17 (31) | Not Stated |
| K. Robb et al. | 2019 | | 415 | Not Stated | Not Stated | Not Stated | Not Stated | Not Stated | Not Stated |
| L.J. Schlapbach et al. | 2019 | | 80 | 7.09 | 69 | 47.6 | 4.95 (3.3 – 8.1) | 32 (40) | 41 (51) |

Abbreviations: P: Peripheral Cannulation. C: Central Cannulation. N: Neonates. P: Pediatric Patients

Supplementary Table 5: Egger’s Test Result for publication bias

| Groups | P-Value^a^ | P-Value^b^ |
| --- | --- | --- |
| Total ECMO | <0.05 | 0.28 |
| VA ECMO | 0.12 | 0.76 |
| Pediatric Only | 0.78 | - |
| Neonatal Only | 0.73 | 0.89 |

Abbreviations: a: Before removal of influential studies. b: After removal of influential studies.

| Author | Year |  | Question Number | | | | | | | | |
| --- | --- | --- | --- | --- | --- | --- | --- | --- | --- | --- | --- |
|  |  | 1 | 2 | 3 | 4 | 5 | 6 | 7 | 8 | 9 | Overall Score |
| C.W. Lillehei et al. | 1989 | ✓ | ✓ | ✓ | ✓ | ✓ | ✓ | ✓ | ✓ | ✓ | 9 |
| S. McCune et al. | 1990 | ✓ | ✓ | ✓ | ✓ | ✓ | ✓ | ✓ | ✓ | ✓ | 9 |
| J. R. Hocker et al | 1992 | ✓ | ✓ | ✓ | ✓ | ✓ | ✓ | ✓ | ✓ | ✓ | 9 |
| D. Cochrane et al. | 1992 | ✓ | ✓ | ✓ | ✓ | ✓ |  | ✓ | ✓ | ✓ | 8 |
| M. Nagaya et al. | 1993 | ✓ | ✓ | ✓ | ✓ | ✓ | ✓ | ✓ | ✓ | ✓ | 9 |
| J. Beca and W. Butt | 1994 | ✓ | ✓ | ✓ | ✓ | ✓ | ✓ | ✓ | ✓ | ✓ | 9 |
| A.P. Goldman et al. | 1997 | ✓ | ✓ | ✓ | ✓ | ✓ | ✓ | ✓ | ✓ | ✓ | 9 |
| D. K. Luyt et al. | 2004 | ✓ | ✓ | ✓ | ✓ | ✓ | ✓ | ✓ | ✓ | ✓ | 9 |
| G. Maclaren et al. | 2007 | ✓ | ✓ | ✓ | ✓ | ✓ | ✓ | ✓ | ✓ | ✓ | 9 |
| R. Tiruvoipati et al. | 2007 | ✓ | ✓ | ✓ | ✓ | ✓ | ✓ | ✓ | ✓ | ✓ | 9 |
| S. J. Wu et al. | 2007 | ✓ | ✓ | ✓ | ✓ | ✓ |  | ✓ | ✓ | ✓ | 8 |
| S. Horton et al. | 2010 | ✓ | ✓ | ✓ | ✓ | ✓ | ✓ | ✓ | ✓ | ✓ | 9 |
| G. Maclaren et al. | 2011 | ✓ | ✓ | ✓ | ✓ | ✓ | ✓ | ✓ | ✓ | ✓ | 9 |
| C. C. Peng et al. | 2012 | ✓ | ✓ | ✓ | ✓ | ✓ |  | ✓ | ✓ | ✓ | 8 |
| Y. Kawai et al. | 2015 | ✓ | ✓ | ✓ | ✓ | ✓ |  | ✓ | ✓ | ✓ | 8 |
| J. Rambaud et al | 2015 | ✓ | ✓ | ✓ | ✓ | ✓ |  | ✓ | ✓ | ✓ | 8 |
| A. Ruth et al. | 2015 | ✓ | ✓ | ✓ | ✓ | ✓ | ✓ | ✓ | ✓ | ✓ | 9 |
| K. Y. Chen et al. | 2016 | ✓ | ✓ | ✓ | ✓ | ✓ |  | ✓ | ✓ | ✓ | 8 |
| A. Sole et al. | 2018 | ✓ | ✓ | ✓ | ✓ | ✓ | ✓ | ✓ | ✓ | ✓ | 9 |
| F. Oberender et al. | 2018 | ✓ | ✓ | ✓ | ✓ | ✓ |  | ✓ | ✓ | ✓ | 8 |
| T. H. Chang et al. | 2018 | ✓ | ✓ | ✓ | ✓ | ✓ |  | ✓ | ✓ | ✓ | 8 |
| K. Robb et al. | 2019 | ✓ | ✓ | ✓ | ✓ | ✓ | ✓ | ✓ | ✓ | ✓ | 9 |
| L.J. Schlapbach et al. | 2019 | ✓ | ✓ | ✓ | ✓ | ✓ | ✓ | ✓ | ✓ | ✓ | 9 |
|  |  |  |  |  |  |  |  |  |  |  |  |

Supplementary Table 6: Outcomes of the Joanna Briggs Institute Checklist for prevalence studies.


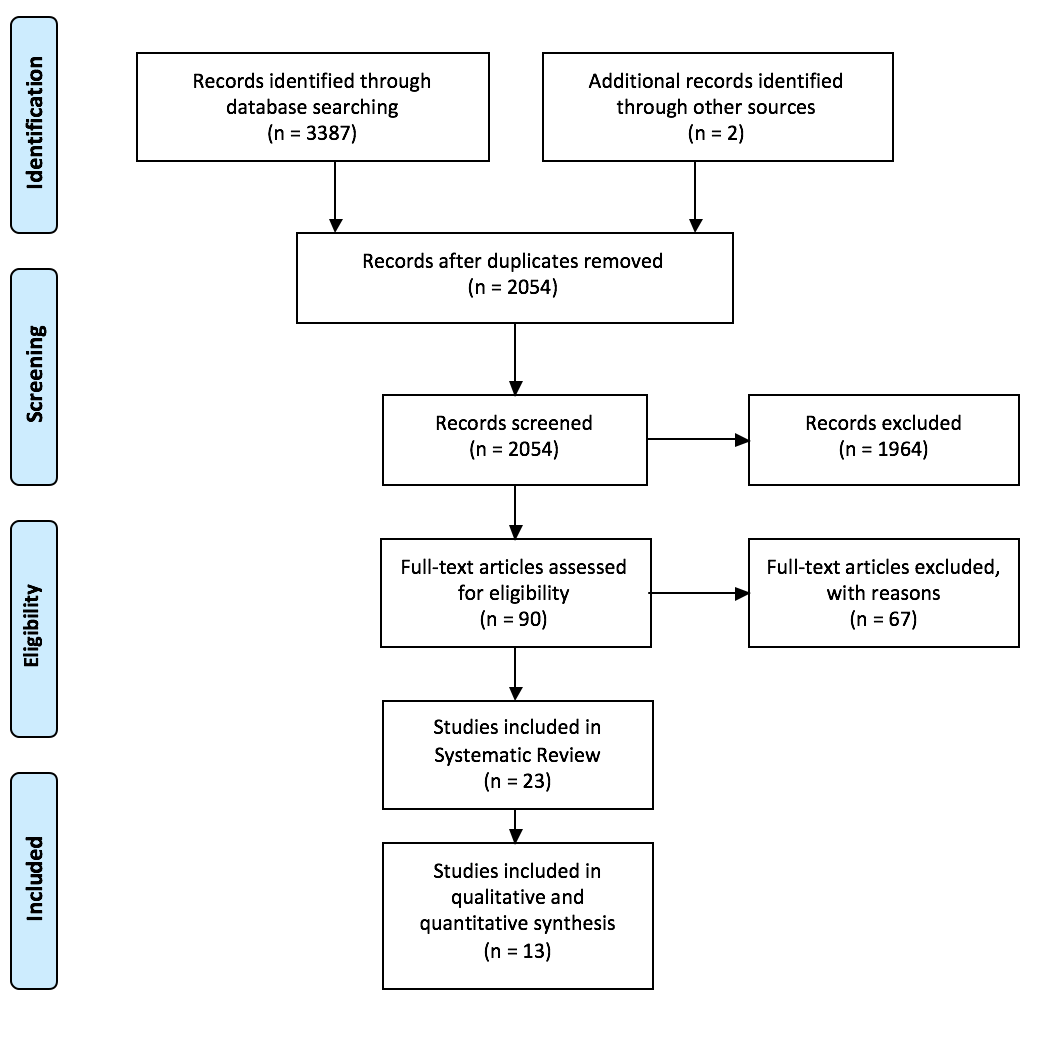


**Supplementary Figure 1**: PRISMA flowchart for study selection

Supplementary Figure 2: Forest plot of studies reporting on children with sepsis needing ECMO after removal of influential studies

**
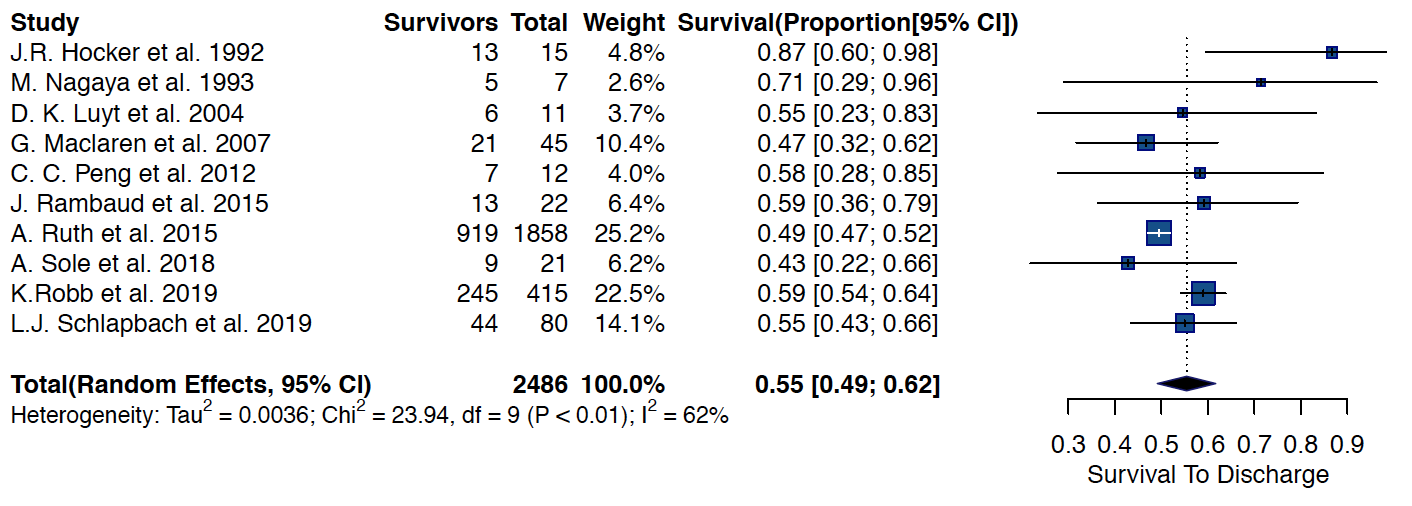
**

Supplementary Figure 3: Forest plot of studies reporting on children with sepsis needing ECMO published from North America


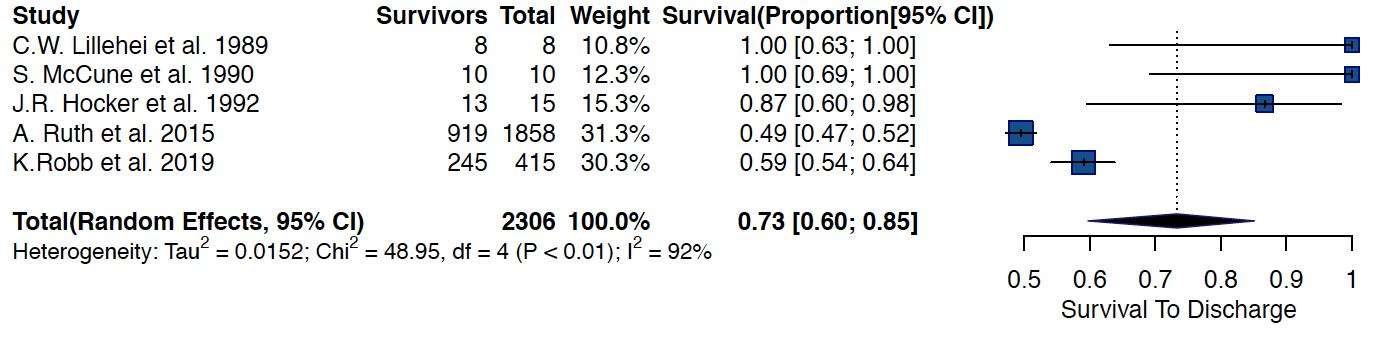


Supplementary Figure 4: Forest plot of studies reporting on children with sepsis needing ECMO published from Australasia


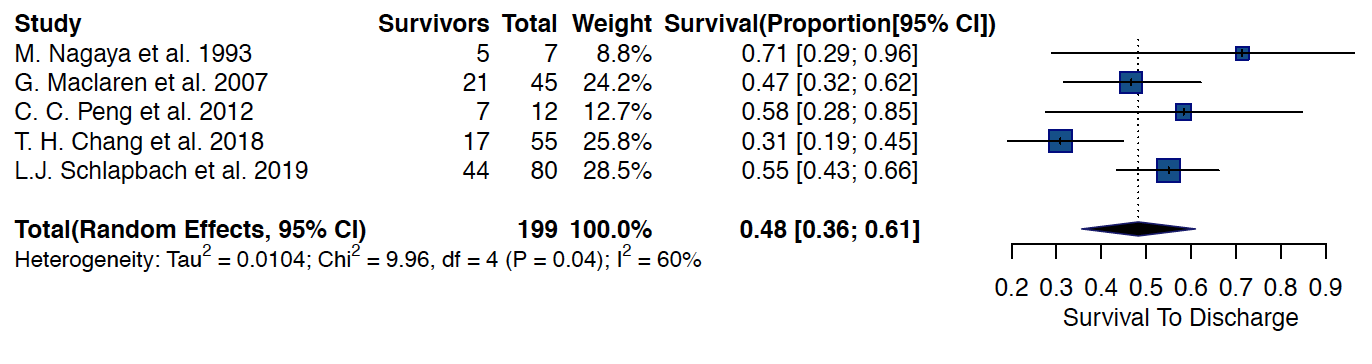


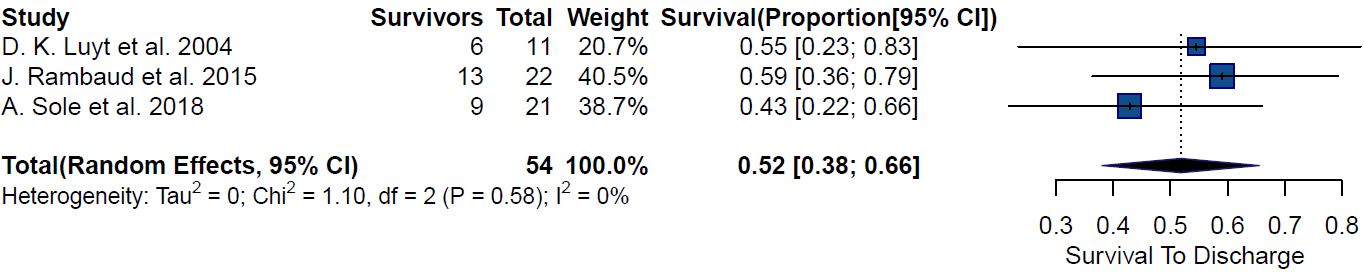
Supplementary Figure 5: Forest plot of studies reporting on children with sepsis needing ECMO published from Europe

Supplementary Figure 6. Forest plot of studies reporting on outcomes of venoarterial ECMO in children with sepsis after removal of influential studies


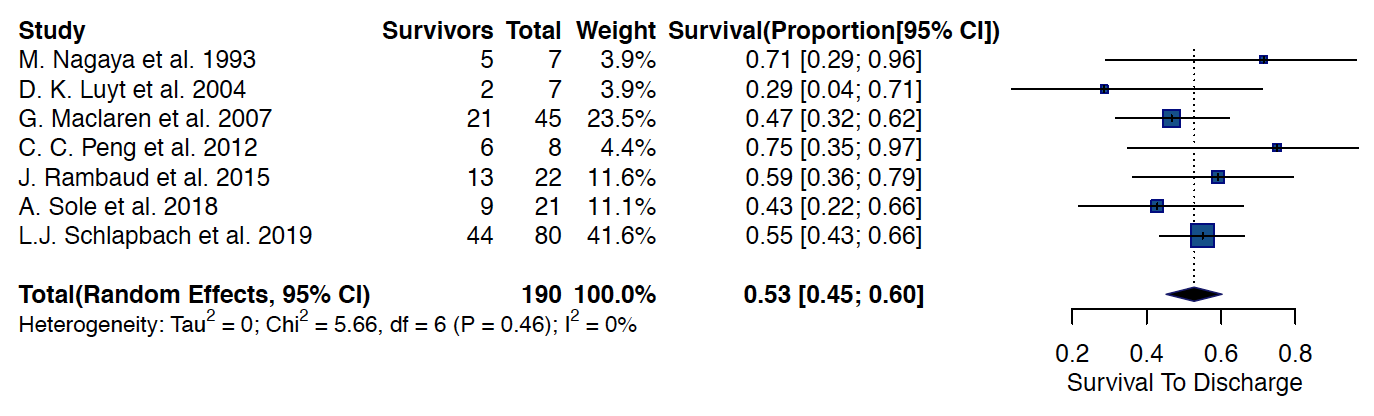


Supplementary Figure 7a and 7b. Forest plot of studies reporting on outcomes in Neonatal patients with sepsis needing ECMO before and after removal of influential studies

7a.


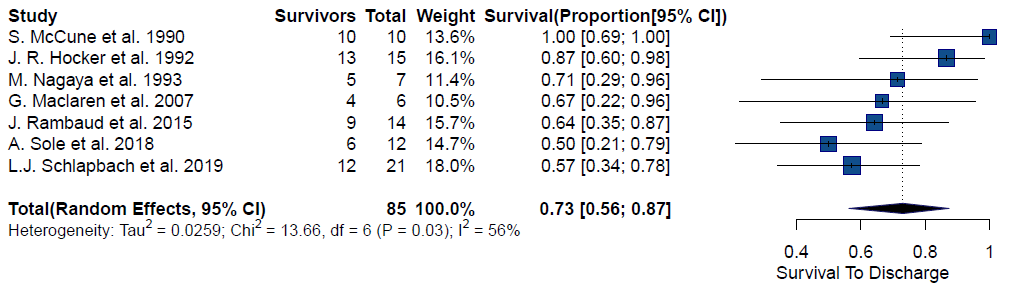


7b.


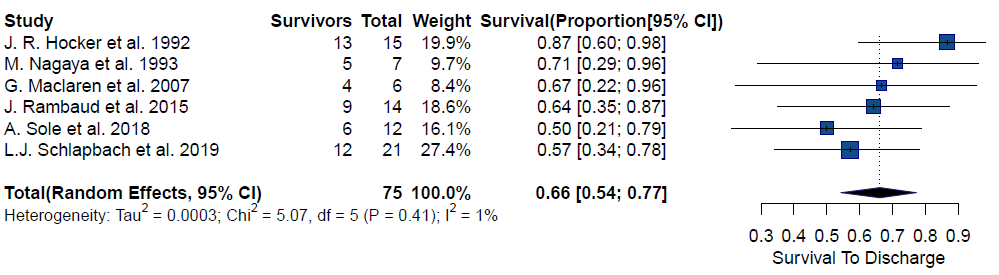


Supplementary Figure 8: Meta-regression plot on Lactate against survival to discharge

Supplementary Figure 9: Meta-regression plot on Length of stay in hospital against survival to discharge

Supplementary Figure 10: Meta-regression plot on Length of stay in ICU against survival to discharge

Supplementary Figure 11: Meta-regression plot on Ecmo Duration against survival to discharge

Supplementary Figure 12: Meta-regression plot on % CPR against survival to discharge

Supplementary Figure13: Meta-regression plot on Renal Replacement Therapy against survival to discharge

Supplementary Figure 14: Funnel Plot before (Left) and after (Right) removal of studies, and Baujat plot of studies that reported on children with sepsis needing ECMO


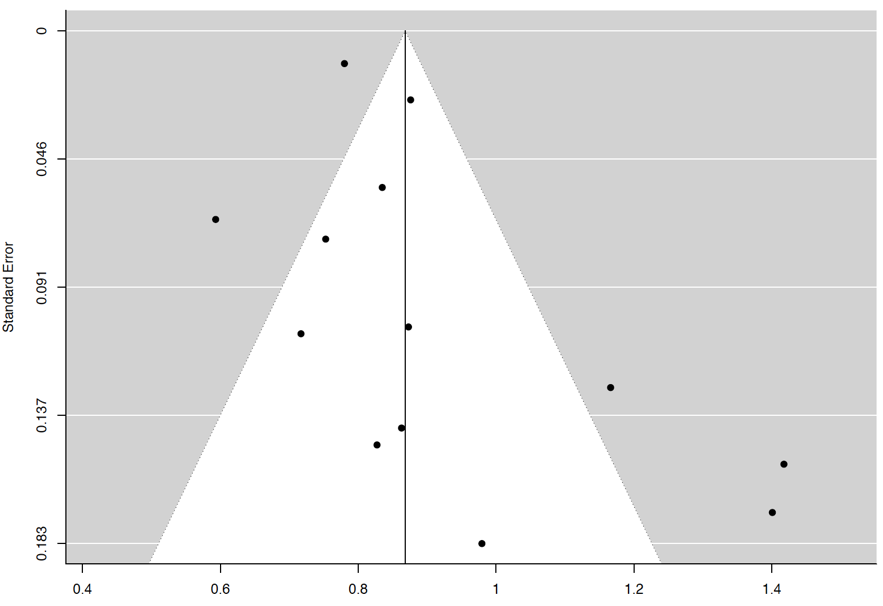

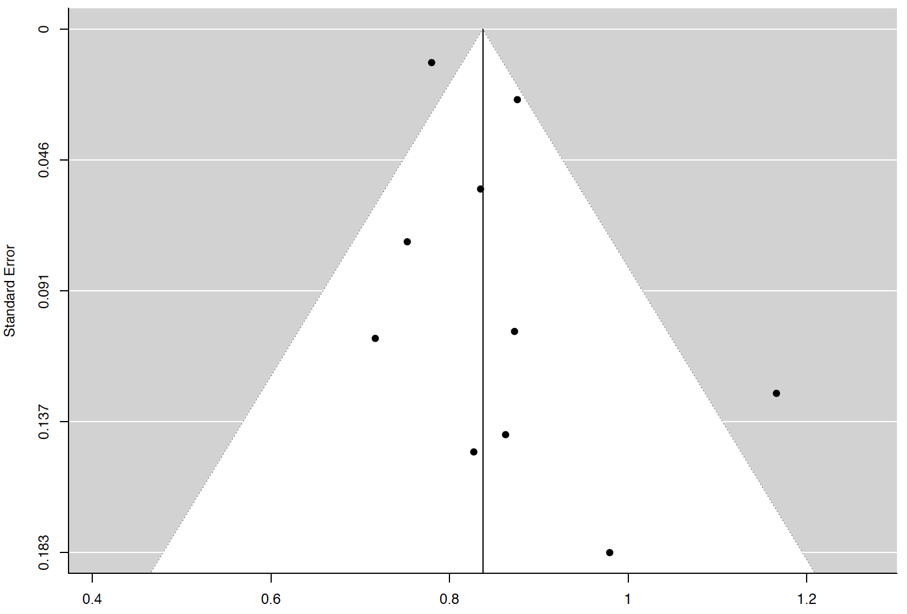


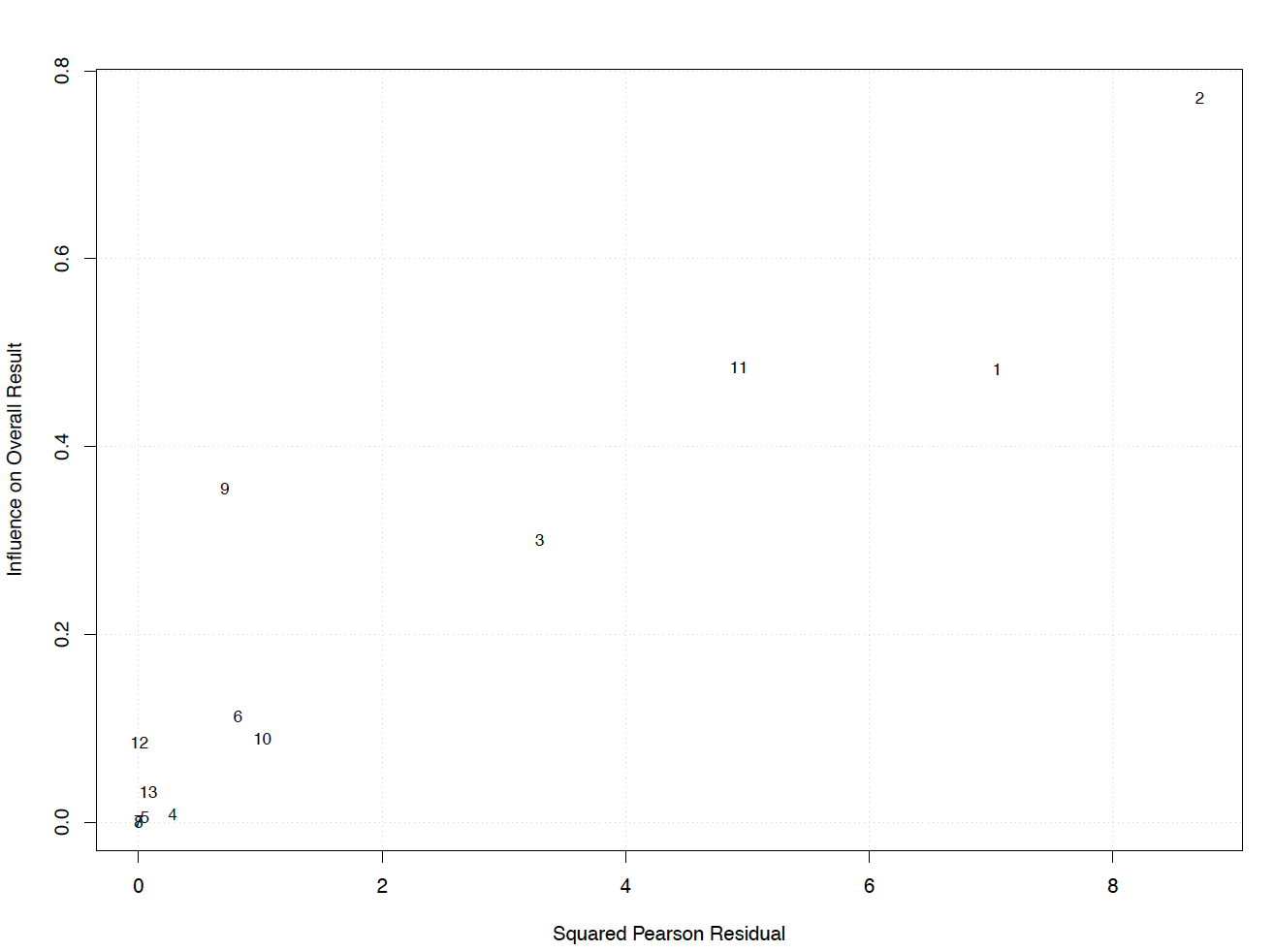


Studies included : 1 C.W. Lillehei et al. 2 S. McCune et al 3: J.R. Hocker et al. 4: M. Nagaya et al. 5: D. K. Luyt et al. 6: G. Maclaren et al. 7: C. C. Peng et al. 8: J. Rambaud et al. 9: A. Ruth et al.. 10: A. Sole et al. 11: T. H. Chang et al. 12: K. Robb et al. 13: L.J. Schlapbach et al.

Supplementary Figure 15: Funnel Plot before (Left) and after (Right) removal of studies, and Baujat plot of studies reporting on patients needing venoarterial ECMO


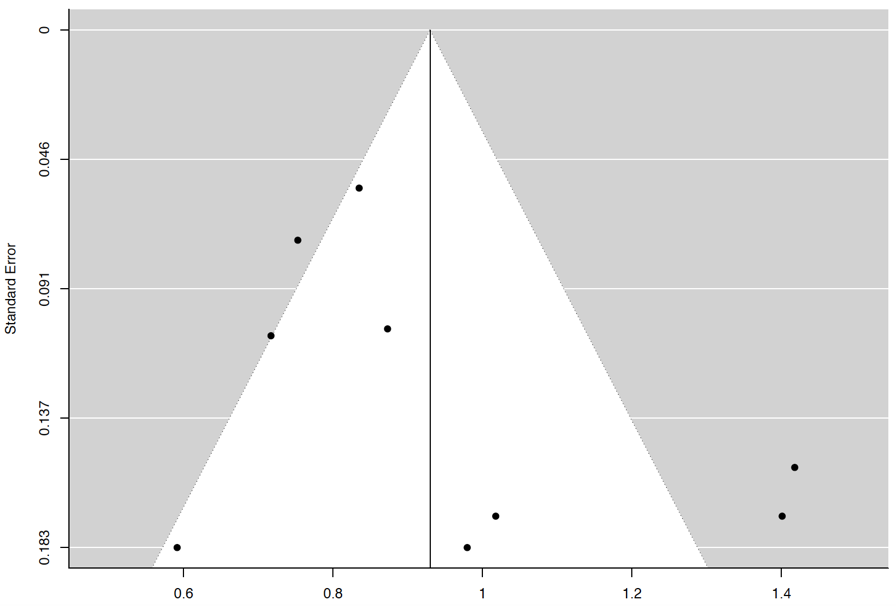

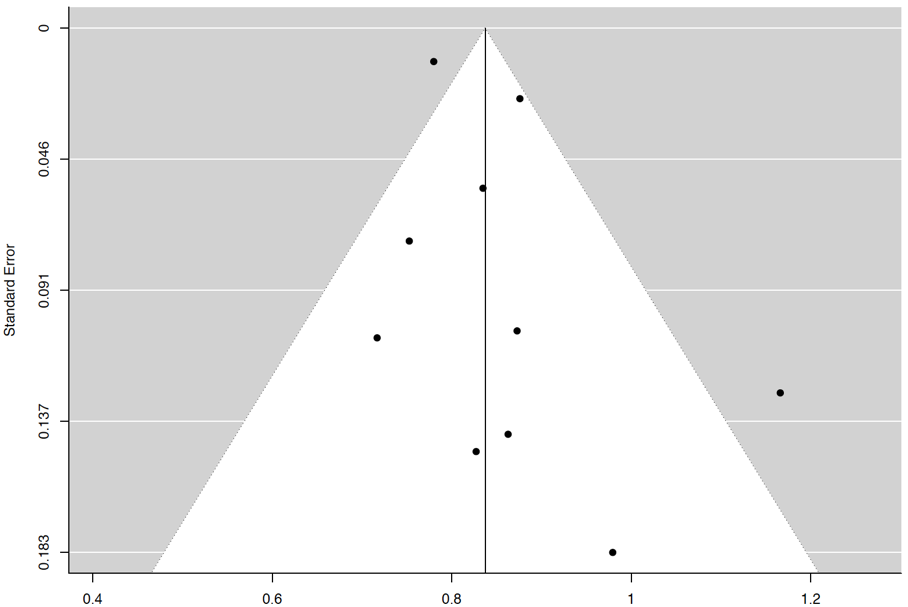


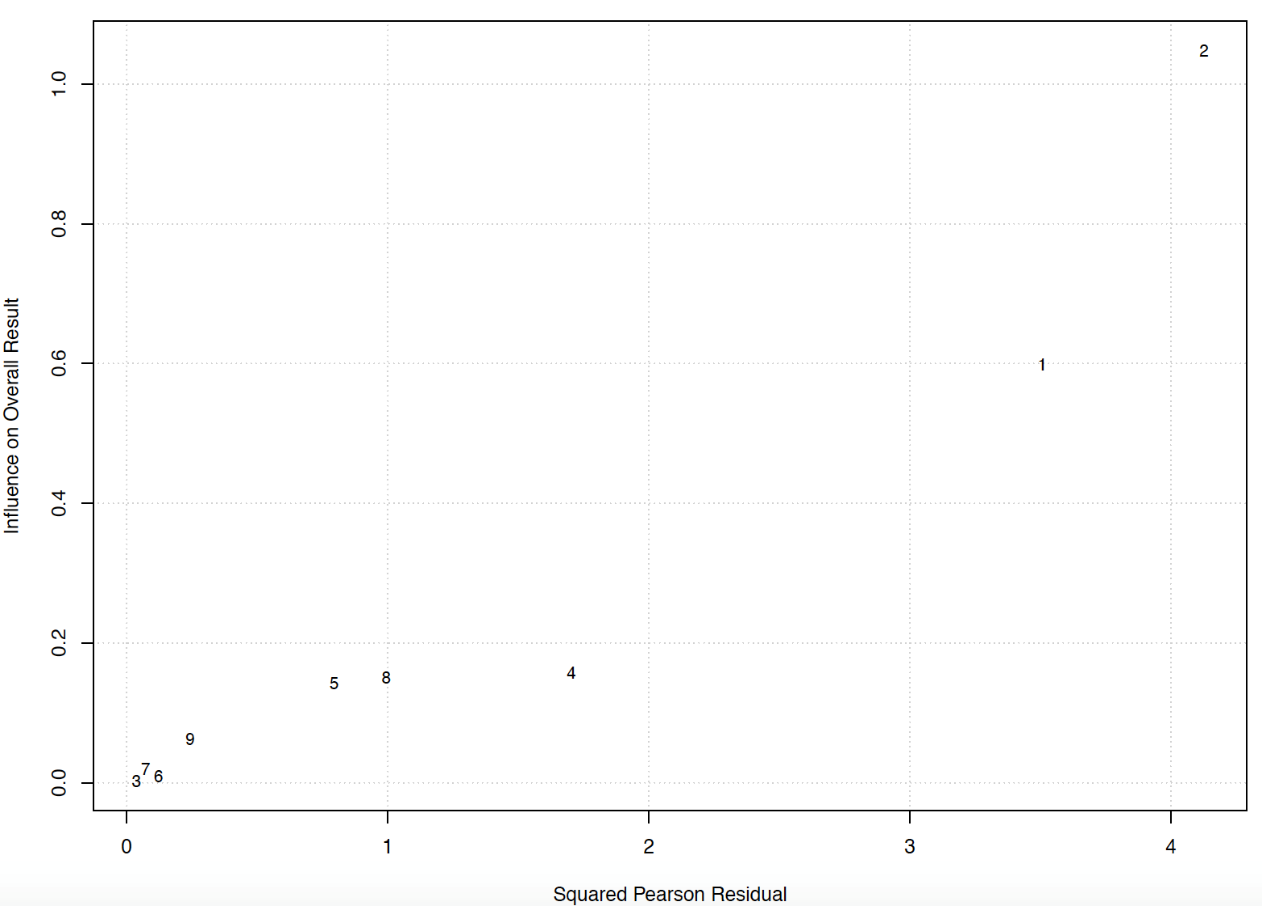


Abbreviations: 1: C.W. Lillehei et al. 2: S. McCune et al. 3: M .Nagaya et al. 4: D.K .Luyt et al. 5: G. Maclaren et al. 6: C.C .Peng et al. 7: J. Rambaud et al. 8. A. Sole et al. 9 L.J. Schlapbach et al.

Supplementary Figure 16: Funnel Plot of Pediatric septic patients


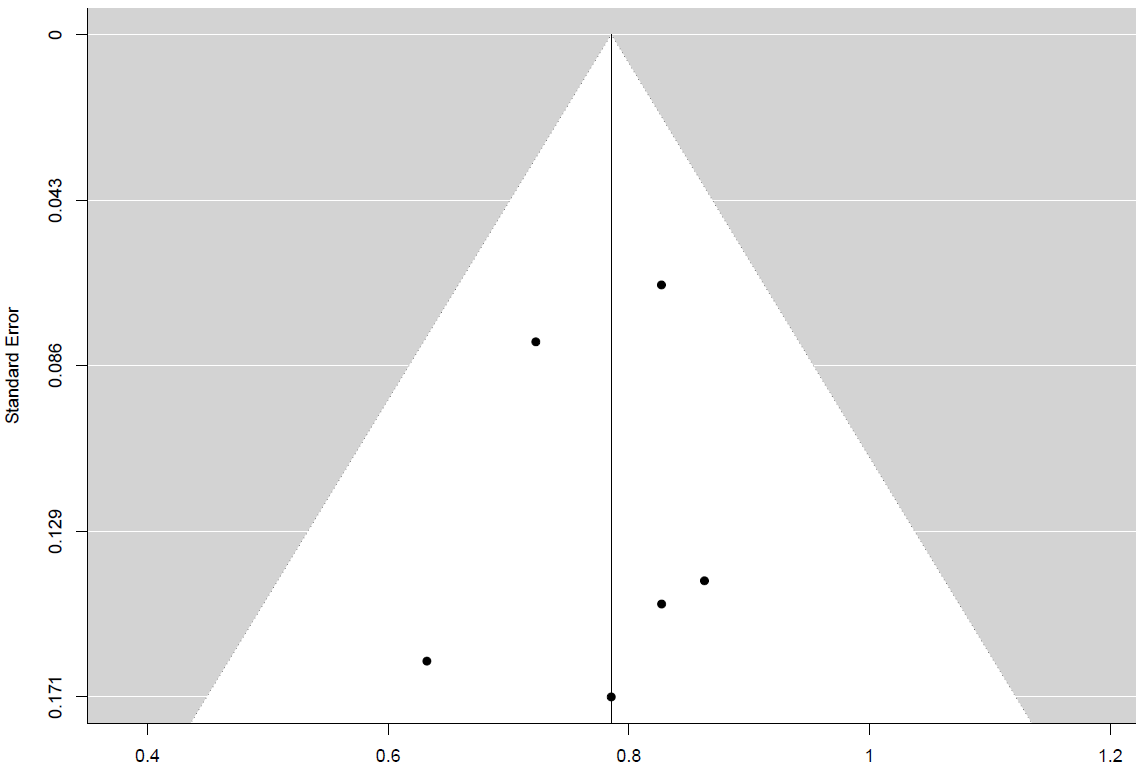


Supplementary Figure 17: Funnel Plot before (Left) and after (Right) removal of studies, and Baujat plot of studies reporting on Neonates with sepsis


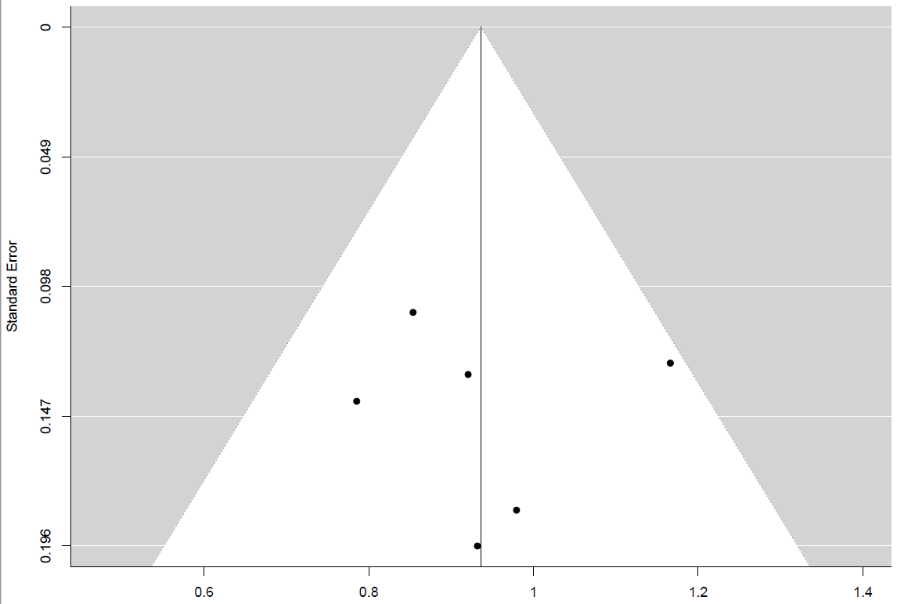

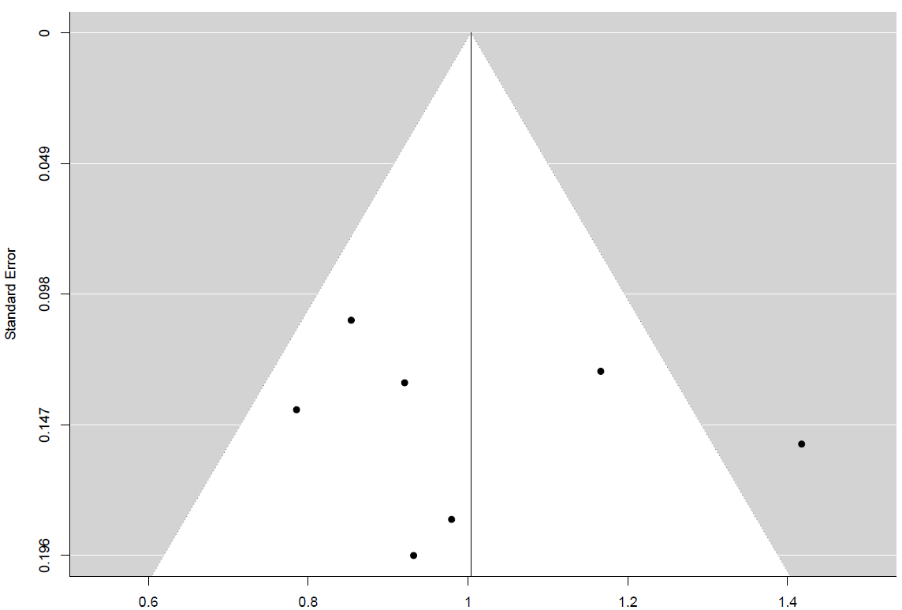


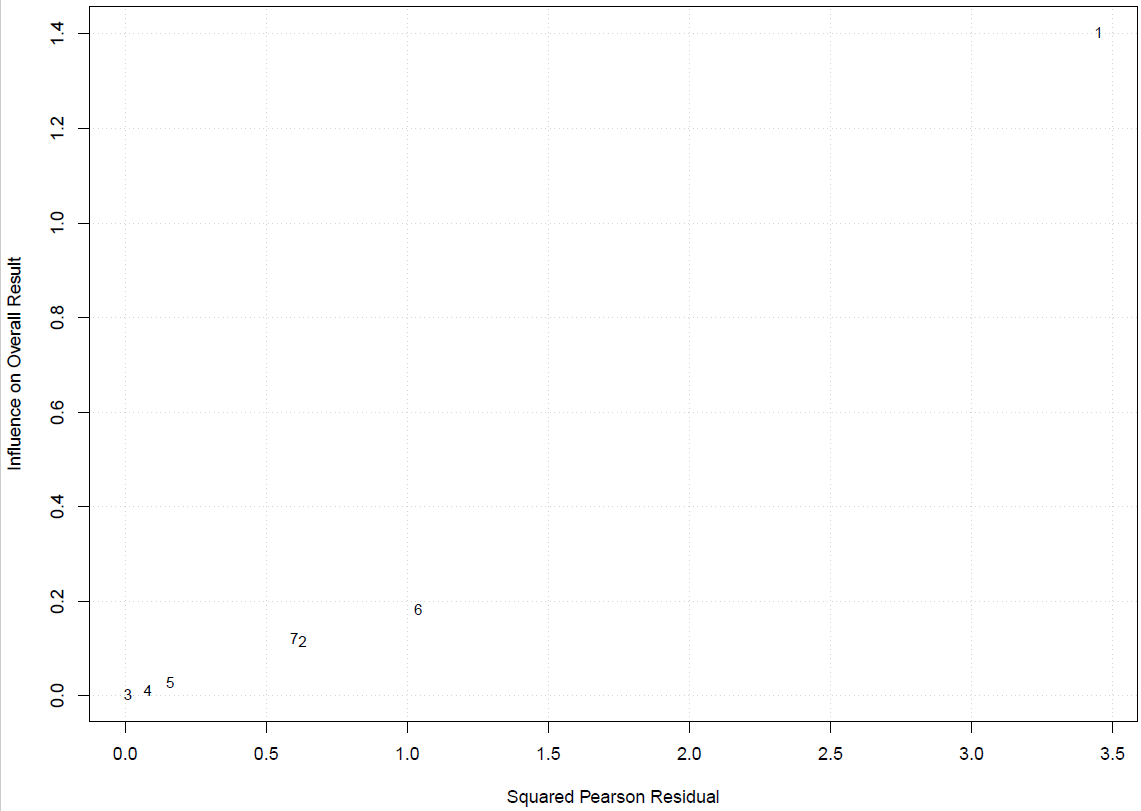


Abbreviations: 1: S .McCune et al. 2. J. R. Hocker et al. 3: M. Nagaya et al. 4. G. Maclaren et al. 5: J .Rambaud et al. 6. A.Sole et al. 7. L.J. Schlapbach et al.
